# Proficiency testing for identifying underperforming students before postgraduate education: a longitudinal study

**DOI:** 10.1186/s12909-020-02184-4

**Published:** 2020-08-10

**Authors:** Vasiliki Andreou, Jan Eggermont, Guy Gielis, Birgitte Schoenmakers

**Affiliations:** 1 Department of Public Health and Primacy Care, Academic Center for General Practice, KU Leuven, Kapucijnenvoer 33 Blok j-Box 7001, 3000 Leuven, Belgium; 2 Department of Cellular and Molecular Medicine, KU Leuven, 3000 Leuven, Belgium; 3 Interuniversity Center for GP Training, 3000 Leuven, Belgium

**Keywords:** General practice, Medical education, Postgraduate education, School admission, Proficiency testing, Validity

## Abstract

**Background:**

Efficient selection of medical students in GP training plays an important role in improving healthcare quality. The aim of this study was to collect quantitative and qualitative validity evidence of a multicomponent proficiency-test for identifying underperforming students in cognitive and non-cognitive competencies, prior to entering postgraduate GP Training. From 2016 to 2018, 894 medical GP students in four Flemish universities in Belgium registered to take a multicomponent proficiency-test before admission to postgraduate GP Training. Data on students were obtained from the proficiency-test as a test-score and from traineeship mentors’ narrative reports.

**Results:**

In total, 849 students took the multicomponent proficiency-test during 2016–2018. Test scores were normally distributed. Five different descriptive labels were extracted from mentors’ narrative reports based on thematic analysis, considering both cognitive and non-cognitive competences. Chi-square tests and odds ratio showed a significant association between students scoring low on the proficiency–test and having gaps in cognitive and non-cognitive competencies during GP traineeship.

**Conclusion:**

A multicomponent proficiency-test could detect underperforming students prior to postgraduate GP Training. Students that ranked in the lowest score quartile had a higher likelihood of being labelled as underperforming than students in the highest score quartile. Therefore, a low score in the multicomponent proficiency-test could indicate the need for closer guidance and early remediating actions focusing on both cognitive and non-cognitive competencies.

## Background

In medicine, school admissions have been the center of attention among medical educators. Successful selection of medical students is of economic, ethical, and societal importance. High quality of medical selection yields highest impact on people’s health and improvement of healthcare quality. This necessity becomes even more apparent in General Practice. The recent societal changes have particularly influenced the profession of General Practitioners (GPs). GPs serve an important role to society as professionals in primary care. More patient-centered decision-making along with increasing multi-morbidity constitutes GPs as patient advocates in primary care.

GP postgraduate education and training seem to differ across Europe [1]. In some European countries, participation in a specific GP training is not required before accreditation as a GP. In other countries, medical students have to follow a GP specialist training, but GP curricula greatly differ across Europe. Furthermore, GP specialist training largely takes place in a hospital setting, which is fundamentally different from a typical GP’s workplace. These particularities of General Practice plea for rigorous selection methods [2].

Postgraduate selection procedures in General Practice are also divergent [3–5]. Traditionally, prospective trainees tend to be selected based on their academic attainment. Cognitive competencies and knowledge testing are an inextricable part of medical competence [6]. Nevertheless, previous academic performance seems to be only a good predictor of achievement in early medical education, but it accounts only for 6% of the variance in postgraduate medical education [7]. Thus, the need for considering non-cognitive competencies becomes apparent in postgraduate medical education. Situational judgement tests (SJT) are increasingly used to assess non-cognitive competencies as a selection method [8]. The use of SJTs is globally expanding in medical professions. In Belgium, SJTs are used as an admission tool in undergraduate medical education [9].

SJTs are a reliable way for measuring professional attributes (such as ethical judgement, empathy, integrity, and problem solving) that are important in a wide range of health professions, including General Practice [5]. Designed appropriately, SJTs are reliable, valid, and fair assessment methods of non-academic traits. SJTs are most often presented as hypothetical scenarios (written or video-based), and the students are called to respond on this situation. Although SJTs have been found to have good levels of criterion and incremental validity in the context of healthcare education, their construct validity is highly dependent on specific constructs [10, 11].

Furthermore, the need for social accountability has pushed for incorporating Evidence-Based Medicine (EBM) into medical curricula. EBM teaching has been integrated into postgraduate medical education and assessment [12]. Research shows that knowledge, skills, and attitudes in EBM are best measured together, rather than separately [13].

Given the importance of successful selection of medical students, we hypothesize that a proficiency-test comprising knowledge testing, SJTs, and EBM could efficiently detect students that underperform in cognitive and non-cognitive competencies before entering postgraduate medical education. The importance of this study lays in detecting underperforming medical students based on a more holistic view of performance. This article presents a validity study of a multicomponent proficiency-test to identify underperforming students prior to postgraduate GP Training.

Following the most recent evolutions around validation, we adopted an argument-based approach to validation [14]. In this line of thought, validity is seen as collecting evidence to support the interpretation and use of the test scores [15–17]. In our study, validity evidence assisted to evaluate the plausibility of the interpretation and usefulness of the proficiency-test [14, 17]. There are five types of different validity evidence: content, internal structure, relationship with other variables, response process, and consequences [17–20]. What follows is a presentation of evidence about the relationship of the test scores with other variables, specifically mentors’ narrative indicators for performance. This study is a follow-up study to the original work published by Schoenmakers and Wens [20]. Validity evidence about the content and the internal structure of the test is presented in Schoenmakers and Wens (content: blueprint of the test items, ensuring variance in item sampling, item development by an expert panel; internal structure: internal consistency based on Cronbach’s alpha and Gaussian distribution) [20].

## Methods

### Setting

According to Flemish law, universities are allowed to set their own admission requirements for postgraduate medical education. Therefore, in collaboration with the four Flemish Universities (KU Leuven, UGent, UAntwerpen and VUB), a three-phase admission procedure was established in 2016 for the GP Training. Phase 1 is administrative, and it stipulates that the candidate must hold a master’s degree in Medicine, must have completed a 6 weeks traineeship in General Practice, should be enrolled at a Flemish University, and should fluently speak Dutch. Phase 2 includes taking a machine-assisted, multicomponent proficiency-test, while phase 3 refers to an evaluation by an interuniversity jury committee of the candidates who failed phase 2. Students who pass the test can only follow the GP Training. This admission procedure and the curriculum are regulated by the Interuniversity Center for GP Training.

The proficiency-test comprises three components; the first component assesses knowledge, the second addresses EBM skills, and the third one is based on SJT. To tackle the large numbers of applicants, a machine-assisted test setting was chosen. Students were already familiar with the online test environment from previous curricular exams. To ensure and enhance test reliability, the test questions were constructed as multiple-choice.

The design of the proficiency-test is discussed more extensively in Schoenmakers and Wens [20]. The results of the proficiency-test are not binding; however, students receive feedback for further development and remediation. After taking the test, students are paired with mentors that support them throughout their traineeship. We use the term “mentors” to refer to workplace-based trainers and university-based trainers. Workplace-based trainers are eligible to choose their trainees through interviews, while the Interuniversity Center for GP Training appoints university-based trainers who support a group of students (approximately 10 students per group) at the university. All mentors have received training for their roles, with providing feedback as a recurring theme in the training sessions. Mentors had no knowledge about students’ performance on the proficiency test.

### Participants

In total, 894 final-year-master students registered to take the proficiency-test during the period 2016–2018 in Flanders. A former specialized training in General Practice was not required. We separated the students in cohorts depending on the year of taking the proficiency-test (2016, 2017, and 2018).

### Data collection

To pursue the aims of the study, we employed a longitudinal cohort design. We gathered data from 2016 to 2018 both in a quantitative and in a qualitative way to collect validity evidence. To extract quantitative data, we used the proficiency-test scores (total test scores as percentages) from 2016 to 2018, while we gathered qualitative data through mentors’ narrative reports during the first year of students’ traineeship.

Mentors gather and report information regarding students’ performance deriving from workplace-based assessments. Workplace-based learning is the basis for the GP Training, while students also receive support from the university-based trainers by participating in peer groups. Moreover, students have to complete five Direct Observation of Procedural Skills (DOPS) at the workplace, along with three monthly meetings with their appointed group. After each evaluation moment, mentors have to provide information as a score and in a free text to the Interuniversity Center for GP Training about students’ performance, using the CanMEDS roles as guideline.

### Data analysis

We divided the students into four score quartiles based on their total test score in ascending order (starting from quartile 1 with the students having performed the lowest). By doing so, particular attention was paid to students that scored high and low on every component of the proficiency test. In addition, we evaluated what the risk was for students who performed in the lowest quartile of the proficiency-test having problems in practice. Data from mentors’ narrative reports were thematically analyzed and coded focusing on both cognitive and non-cognitive competencies [21]. The thematic analysis of the qualitative data was done by two researchers (VA and BS) separately. Discrepancies in coding were discussed until consensus was reached and a third researcher (JE) was the external referee, if disagreements arose [22]. Qualitative analysis was performed with the software program QSR International’s NVIVO version 11.

Taking into consideration the thematic analysis, we assigned a descriptive label to the students when necessary. The labels indicated whether students underperformed and which type of competency they were lacking. We used chi-square tests to explore whether there is a relationship between students receiving a label by their mentors and ranking in the highest and lowest score quartiles. Afterwards, we calculated the effect size to estimate the strength of the association between the variables. We analyzed the data using SPSS 25 (IBM SPSS Statistics 25).

## Results

In total, 894 students inscribed to take the proficiency-test in the course of 3 years (2016–2018). Out of these 894 students, 45 were excluded either because they had dropped out without continuing into the GP Training, or did not complete the test. In 2016, 323 medical students took the proficiency-test, while in 2017 and in 2018, there were 305 and 266 candidates respectively. The scores were normally distributed with a mean score in 2016 of approximately 66.92%, and a standard deviation of 7.49%; in 2017, the mean score was 69.23%, and the standard deviation was 4.92%; in 2018, the mean score was 66.85% and the standard deviation was 4.87% (see Table 1).

**Table 1:** Descriptive statistics of total scores of the proficiency-test for the 2016–2018 period

	N	Minimum	Maximum	Mean	Standard Deviation
Total scores 2016	323	36.70%	80.47%	66.96%	7.49%
Total scores 2017	305	39.37%	79.74%	69.23%	4.92%
Total scores 2018	266	47.50%	78.42%	66.85%	4.87%

**Fig. 1:**
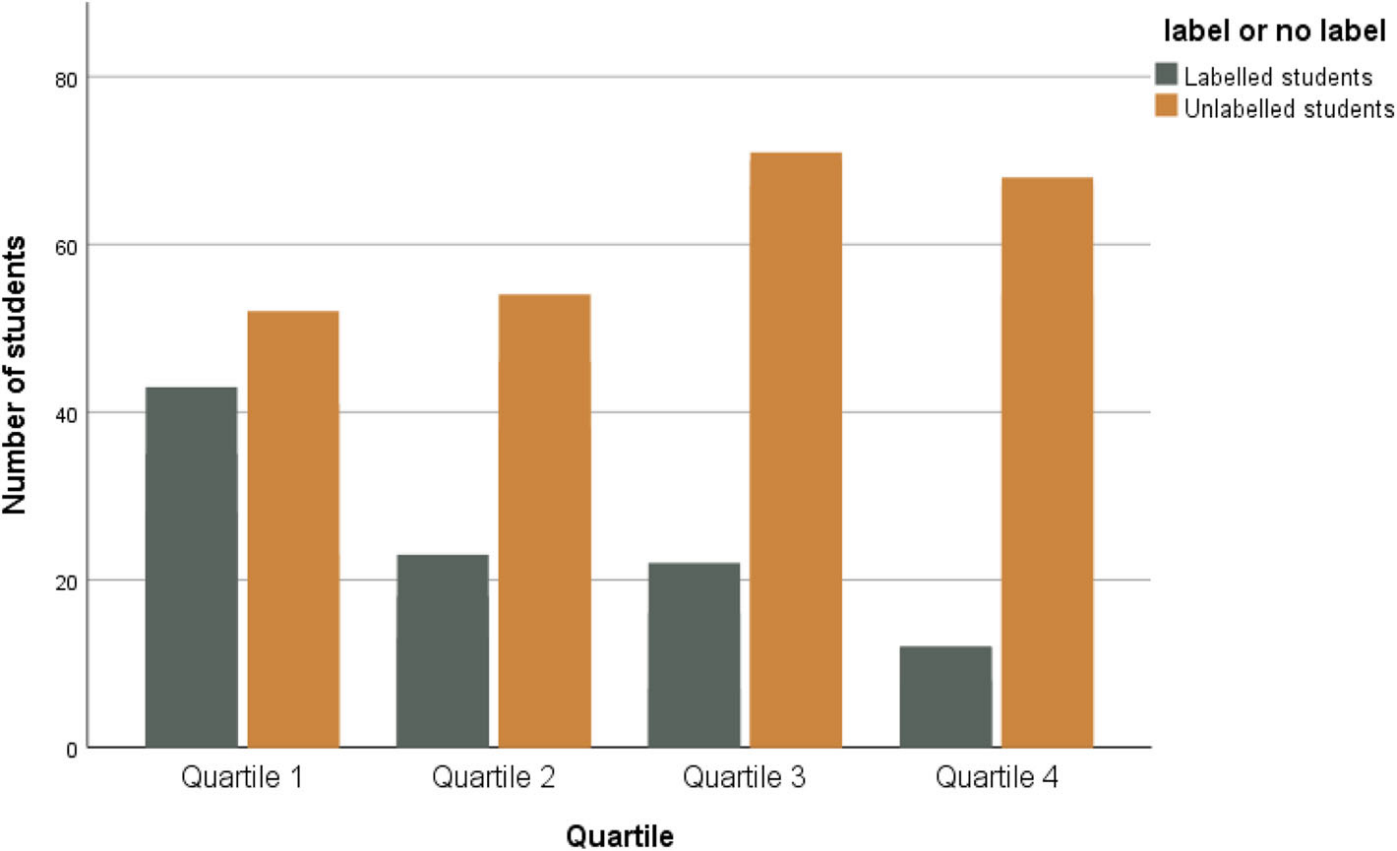
Distribution of students with and without a label per score quartile (2016–2018)

**Fig. 2:**
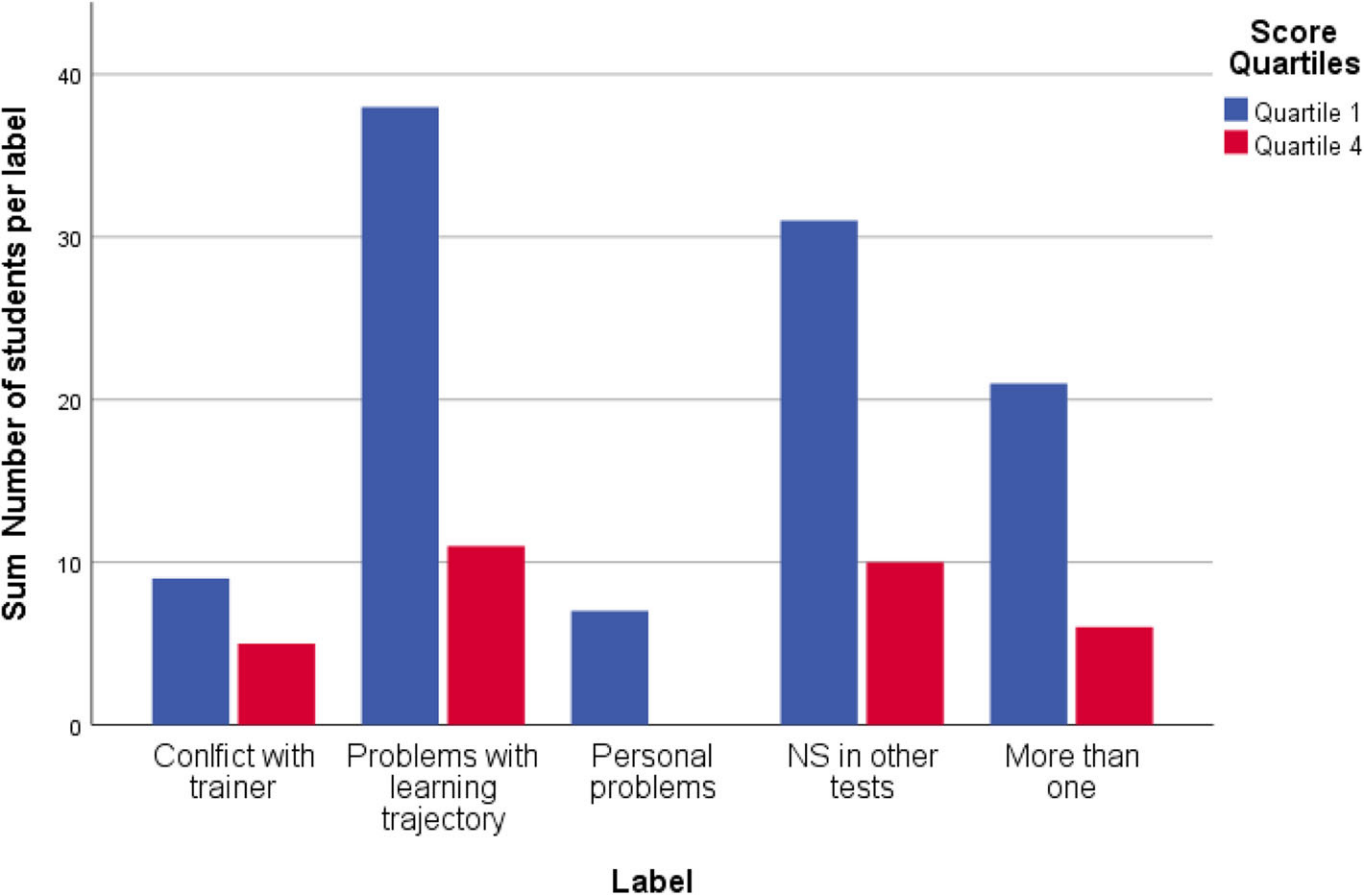
Distribution of students per label within score quartile 1 and score quartile 4 (2016–2018)

Five labels could be discerned in the qualitative data considering both cognitive and non-cognitive competencies. We defined trainees’ medical knowledge as cognitive competencies, while professional attitudes, challenges with self-directed learning, communication with trainer, inhibiting issues for learning in trainees’ life, and learning disabilities as non-cognitive competencies. Three labels related to non-cognitive competencies. First label was ‘conflict with trainer’; this refers to conflicts arising between trainee and trainer (cultural differences, different expectations, lack of attitude, etc.) Second label was ‘problems with learning trajectory’ to refer to students that faced challenges with self-directed learning; the mentors labelled some students as not consequent with self-study, following deadlines and attending seminars. Third recurring label was ‘personal problems’ referring to trainee’s psychological issues, learning difficulties (ADHD, autism, etc.), and problems in trainees’ private life that might influence their performance. The fourth label, which focused on cognitive competencies, was ‘Not Succeeded in other tests’ and it refers to students that passed the proficiency-test, but they failed other curriculum assessments. Last label was ‘more than one’ signaling students with multiple problems. In total, 237 students were labelled. Figure 1 illustrates the number of students with and without a label per score quartile, while Fig. 2 provides an overview of students’ distribution per label within score quartile 1 and 4. The fact that a large number of students did not receive a label means that the mentors did not detect any crucial problems during students’ first year in the postgraduate GP Training.

In 2016, quartile 1 included 80 students out of 323 participants. Out of 80 students, 28 were labelled. More specifically, three students were labelled as ‘conflict with trainer’ and three students as ‘personal problems’*;* twelve students had failed another test while four students were reported to have ‘more than one’ problems. Quartile 4 included 79 students and twelve out of 79 were labelled. Two students had a ‘conflict with their trainer’; four students were experiencing ‘problems with their learning trajectory’; four students had failed in other tests, and two students had multiple problems.

In 2017, 76 students out of 305 scored in quartile 1, and 76 also scored in quartile 4. In quartile 1, 35 students received a label. Of these 35 students, two students were labelled with ‘personal problems’, five students had a ‘conflict with their trainer’, while eight students were labelled as facing ‘problems with their learning trajectory’; twelve students had not succeeded in other assessments, and eight students were falling under ‘more than one’ category. In quartile 4, eight students were labelled. Three students had ‘problems with their learning trajectory’, and one student had ‘conflicts with their trainer’; two students had failed other curricular exams, and two students were experiencing more than one problems.

In 2018, the number of students in quartile 1 and in quartile 4 was 66 out of 266 respectively. In quartile 1, 43 students were labelled. Specifically, one student had a ‘conflict with their trainer’, and two students had ‘personal problems’; seven students had failed other curriculum tests, while twenty-four students were having ‘difficulties with their learning trajectory’; nine students faced multiple problems. In quartile 4, twelve students were labelled. Two students had ‘conflict with their trainer’, four students were having ‘difficulties with their learning trajectory’, while four others had failed other assessments; two students were experiencing different problems at the same time.

For every year the proficiency-test took place, different chi-square tests were performed. Significant results were found for every test year (see Table 2). More specifically, in 2016, there was a significant association between total score quartiles and whether students were labelled χ^2^ (1, *N* = 159) = 8.29, *p* < 0.004 (see Table 2). The odds ratio showed that the odds of students being labelled was almost 3 times higher if they had obtained a low total score (see Table 3). The percentage of students that were labelled also significantly differ by score quartile in 2017, χ^2^ (1, *N =* 159) = 23.64 *p* < 0.001 (see Table 2). The odds of students being labelled was 7.26 higher, if they were ranked in quartile 1 (see Table 3). The relation between score quartiles and whether students were labelled was significant in 2018 as well, χ^2^ (1, *N* = 132) = 29.95 *p <* 0.001 (see Table 2). The odds ratio showed that the odds of students being labelled was 8.41 higher if they belonged to score quartile 1 (see Table 3).

**Table 2:** Chi-square tests per test year for score quartiles and labels

Chi-square tests 2016–2018			
	Value	df	Asymptotic Significance (2-sided)
Pearson Chi-Square 2016	8.285^a^	1	0.004
Pearson Chi-Square 2017	23.642^a^	1	0.000
Pearson Chi-Square 2018	29.953^a^	1	0.000

**Table 3:** Effect estimate of students ranking in quartile 1 and receiving a label per test year

Risk Estimate			
	Value	95% Confidence Interval	
		Lower	Upper
Odds Ratio for Score Quartiles Test Year 2016 (Quartile 1/ Quartile 4)	3.006	1.396	6.475
Odds Ratio for Score Quartiles Test Year 2017 (Quartile 1/ Quartile 4)	7.256	3.070	17.153
Odds Ratio for Score Quartiles Test Year 2018 (Quartile 1/ Quartile 4)	8.413	3.762	18.813

## Discussion

The study results show that a multicomponent proficiency-test could detect students who were low-performers in cognitive and non-cognitive competencies during their first year of GP Training. The proficiency-test is a part of a three-phase admission procedure for the GP Training in Flanders, Belgium. Students need to prove proficiency and succeed on every component of the test in order to be admitted. Although the test comprises three components, this study aimed at collecting validity evidence of the test as a whole in relationship with other assessments.

Once the students had taken the test, they were paired with mentors that reported on students’ individual progress for the first year of their traineeship. Based on the mentors’ narrative reports, the students were assigned a descriptive label, when they were facing difficulties during their traineeship. The thematic analysis of the reports produced five different descriptive labels. One label was related to underperformance in cognitive competencies, namely ‘NS in other tests’, while three labels identified underperforming students in non-cognitive competencies; the fifth label referred to students with multiple problems. Although the fourth label appears ‘NS in other tests’ different than the other three, it can be explained by the fact that mentors mainly relied on other curricular assessments as to evaluate cognitive competencies.

The thematic analysis also illustrated that the majority of students with labels are situated in the lowest total score quartile (quartile 1). In particular, 103 students out of 849 were labelled and were ranked in score quartile 1. The majority of low performing students seems to have problems with their learning trajectory or have failed other assessments. Most importantly, students facing multiple problems mostly performed low in the proficiency-test. Strikingly, in 2016, 12 students in quartile 4 appeared to experience multiple problems. Most probably, these students needed more support and close monitoring throughout their first year of traineeship.

The chi-square tests and odds ratio also show a significant association between score quartile and whether a student was labelled or not. It is notable that the odds ratio and chi-square results in 2016 are lower than the results of 2017 and 2018. This could be related to the fact that the proficiency-test was for the first time administered in 2016, consequently students were not acquainted yet with the test format.

### Limitations

A limitation of this study is that no demographics of the test participants were collected. Since no specialized medical training is required before taking the proficiency-test, every medical student is allowed to participate. Some students took the test, although they did not wish to follow a postgraduate GP Training. It could be possible that students’ preferences and motivation might play a role in how they perform on the test.

Another limitation would be that the mentors could choose what information seemed important to be communicated with the Interuniversity Center for GP Training. Therefore, the reports unavoidably contain a degree of subjective bias. This study did not also discuss the reliability of the multicomponent proficiency-test, because its main aim was to gather validity evidence. It seems reasonable to examine issues regarding reliability in the future.

Finally, we only analyzed the total score results without taking into account students’ scores on the different components. Thus, we might have missed relevant information to non-cognitive competencies from the SJT and EBM components. Nevertheless, we were only interested in validity evidence of the test in its totality.

## Conclusion

The challenge of what needs to be measured is a persistent problem in medical selection research. Selection methods often focus on cognitive competencies as outcome measures (e.g. performance on medical exams), rather than on non-cognitive. However, outcomes measures should be different when transitioning from undergraduate to postgraduate medical education. This study demonstrates that a multicomponent proficiency test (focusing on knowledge testing, SJTs, and EBM) could detect underperforming students prior to postgraduate GP Training by assessing both cognitive and non-cognitive competencies. The findings suggest that a low score on the proficiency test might imply closer guidance and early remediating actions aiming on both cognitive and non-cognitive competencies. Longitudinal data collection enabled illustrating more the outcome measures, and providing validity evidence regarding the relationship of the proficiency-test with other forms of assessment.

## Data Availability

The datasets used and/or analyzed during the current study are available from the corresponding author on reasonable request.

## Abbreviations

GP: General Practitioner
SJT: Situational Judgement Test
EBM: Evidence-Based Medicine
DOPS: Direct Observation of Procedural Skills
NS: Not Succeeded
